# Chronic Pain: Clinical Updates and Perspectives

**DOI:** 10.3390/jcm11123474

**Published:** 2022-06-16

**Authors:** Carmen M. Galvez-Sánchez, Casandra I. Montoro

**Affiliations:** Department of Psychology, University of Jaén, 23071 Jaén, Spain

The International Association for the Study of Pain (IASP) has defined pain as an unpleasant sensory and emotional experience associated with actual or potential tissue damage, or described in terms of such damage, which also comprises a subjective component [1]. Pain is characterized by a multidimensional nature in which three dimensions are usually differentiated: sensory-discriminative, emotional-affective, and cognitive-evaluative [1]. Furthermore, pain can be classified according to different aspects, for example the duration, the explaining cause, or the anatomical location [1]. Depending on the duration and the main classification, it is possible to differentiate between acute pain (less than 6 months) or chronic pain (more than 6 months) [1].

Regarding chronic pain, it entails a serious health burden, and a high comorbidity with other disorders such as anxiety, depression, insomnia, and cognitive impairments [2,3,4,5,6,7,8,9,10,11]. Given its high comorbidity with the above-reported disorders, chronic pain generates important socio-health expenses related to its management, as well as numerous indirect economic costs as a result of high rates of absenteeism, reduced labor productivity, and disability [12,13]. The prevalence of chronic pain in the general population of developing countries is estimated at 18% [14], and it is expected to increase with population growing and aging which in turn will require more effective prevention, diagnosis, and pain treatment strategies due to the higher associated socio-health and personal costs [15]. Chronic pain negatively affects both the patient and relatives’ quality of life [16,17]. Based on the aforementioned, chronic pain has been recognized as a bioethical issue [18] and different international organizations have declared the access to an adequate pain therapy as a human right [17,19,20].

The principal chronic pain disorders include headache, migraine, and local and generalized musculoskeletal diseases. The last may be further divided into those with inflammatory (i.e., autoimmune or infectious), mechanical (i.e., chronic low back pain), or unknown origin [21]. These disorders have in common the tendency to exhibit periods of remission of symptoms and phases of exacerbation [21].

Fibromyalgia, chronic tension-type headache, migraine, and the temporomandibular disorder are some examples of chronic pain disorders without a clear physiological cause. In all of these disorders, the pain intensity is found not to be proportional to the reported injury [22,23,24,25]. Nonetheless, the most empirically supported hypothesis is the presence of an alteration at central nervous system level [3,26]. The central pain processing mechanisms are proposed to be altered and produce a phenomenon called central sensitization. This phenomenon consists of a great sensitization to pain and excitability of the neurons of the posterior horns of the spinal cord and the reticulo-thalamic-cortical system [27,28]. Additionally, central sensitization implies a neuronal reorganization along with a deficient descending pain modulation system leading to an extreme sensitivity to touch, cold, or heat [29,30,31].

The intervention of chronic pain should be considered from a biopsychosocial perspective. As pointed out before, chronic pain is a condition characterized by physiological and biological correlates, in turn modulated by emotions (including negative beliefs) [4,32,33,34,35], personality factors [36,37,38,39,40], and coping styles [8,41], and related to different psychological behaviors [42]. In addition to these factors, the evolution and prognosis of chronic pain is not only affected by social but also by cultural factors (i.e., socio-economics, social support, educational level, work-related situation, etc.) [37,43]. Consequently, the treatment based on a multidisciplinary approach is shown to be the most effective in chronic pain management [44]. Anesthesiologists, psychiatrists, neurosurgeons, psychologists, and physiotherapists are frequently integrated into multidisciplinary pain teams [42]. Despite the benefits of the multidisciplinary approach in the treatment of chronic pain, it is not fully effective [45]. The treatment of chronic pain is still a current social and sanitary challenge [42], which also require personalized care to improve the health-related quality of the lives of these patients [46,47]. Personalized pain medicine remains a gap which needs to be overcome [48]. The person-centered medicine and personalized medicine in the areas of chronic pain research and management (including the cognitive, physical, affective, and behavioral domains) are necessary [46,49]. Authors point out the need for the fusion of these paradigms into a single new approach with the objective to avoid possible miscommunication, duplication of efforts, and ineffective treatment of chronic pain patients [47].

In general, to contribute to the effectiveness of chronic pain treatment, patients must be informed of the characteristics of the treatment. Thus, they are capable of committing to the treatment and can achieve better results [50]. It is crucial: (1) to establish clear and realistic therapeutic goals to motivate patients and increase their adherence to treatment, (2) to promote a good therapeutic alliance or rapport between the patient and the therapist, (3) to help patients to understand their responsibility in the treatment process, and (4) to ensure they are aware of the relevance of their implication to change the perspective from helplessness and despair to self-efficacy and personal self-control over the disorder [49]. Indeed, the perception of control and self-efficacy are issues to be addressed in any of the chronic pain treatments, because patients usually perceive pain as something uncontrollable, which generates low self-efficacy and hopelessness [51]. By contrast, patients with a higher perception of self-efficacy tend to have less pain, negative emotions, and disability and react better to treatment [51,52]. Therefore, active coping strategies must be promoted. Other elements such as postural and sleep hygiene, a proper diet, and regular and adapted physical exercise are further necessary to include in chronic pain treatment [5,49,53]. In addition, the involvement of patients´ relatives is vital for the success of the intervention, and the reinforcement of new, healthier behaviors and lifestyles on patients [54].

In sum, the chronic pain field requires continuous research in order to improve its prevention, diagnosis, and treatment. Considering the worldwide incidence, and prevalence of chronic pain in developing countries [14], scientists, clinicians, and governments must move forward together to overcome the great treatment challenge that chronic pain entails. This will markedly contribute to improving the health-related quality of life of patients and relatives, as well as to decreasing its socio-economic costs. 
